# Upper respiratory tract disease and associated diagnostic tests of mycoplasmosis in Alabama populations of Gopher tortoises, *Gopherus polyphemus*

**DOI:** 10.1371/journal.pone.0214845

**Published:** 2019-04-05

**Authors:** Jeffrey M. Goessling, Craig Guyer, James C. Godwin, Sharon M. Hermann, Franzisca C. Sandmeier, Lora L. Smith, Mary T. Mendonça

**Affiliations:** 1 Auburn University, Dept. of Biological Sciences, Auburn University, Alabama, United States of America; 2 Alabama Natural Heritage Program, Auburn University, Alabama, United States of America; 3 Department of Biology, Colorado State University Pueblo, Pueblo, Colorado, United States of America; 4 Joseph W. Jones Ecological Research Center, Newton, Georgia, United States of America; Montana State University, UNITED STATES

## Abstract

Upper respiratory tract disease (URTD) in North American tortoises (*Gopherus*) has been the focus of numerous laboratory and field investigations, yet the prevalence and importance of this disease remains unclear across many tortoise populations. Furthermore, much research has been focused on understanding diagnostic biomarkers of two known agents of URTD, *Mycoplasma agassizii* and *Mycoplasma testudineum*, yet the reliability and importance of these diagnostic biomarkers across populations is unclear. Gopher Tortoises (*Gopherus polyphemus*) have experienced significant declines and are currently protected range wide. Geographically, Alabama represents an important connection for Gopher Tortoise populations between the core and periphery of this species’ distribution. Herein, we systematically sampled 197 Gopher Tortoises for URTD across seven sites in south-central and south-eastern Alabama. Plasma samples were assayed for antibodies to *M*. *agassizii* and *M*. *testudineum*; nasal lavage samples were assayed for the presence of viable pathogens as well as pathogen DNA. Lastly, animals were scored for the presence of external symptoms and nasal scarring consistent with URTD. External symptoms of URTD were present in *G*. *polyphemus* in all sites sampled in Alabama. There was no relationship between active symptoms of URTD and *Mycoplasma* antibodies, however the presence of URTD nasal scarring was positively related to *M*. *agassizii* antibodies (P = 0.032). For a single site that was sampled in three sequential years, seroprevalence to *M*. *agassizii* significantly varied among years (P < 0.0001). *Mycoplasma agassizii* DNA was isolated from four of the seven sites using quantitative PCR, yet none of the samples were culture positive for either of the pathogens. An analysis of disease status and condition indicated that there was a significant, positive relationship between the severity of URTD symptoms and relative body mass (P < 0.05). This study highlights the need for continued monitoring of disease in wild populations. Specifically, focus must be placed on identifying other likely pathogens and relevant biomarkers that may be important drivers of URTD in North American tortoises. Special consideration should be given to environmental contexts that may render wild populations more susceptible to disease.

## Introduction

Infectious diseases are of increasing risk to the fitness of ectothermic vertebrates [1] especially in light of recent global change [2]. Among North American ectotherms, upper respiratory tract disease (URTD) is one of the most well-studied diseases that affects wild populations of tortoises in the genus *Gopherus* (e.g. [3, 4,5]). Two pathogens have been identified as causative agents of URTD in *Gopherus*, *Mycoplasma agassizii* and *Mycoplasma testudineum* [3, 6]), and enzyme-linked immunosorbent assays (ELISA) have been developed to diagnose the presence of antibodies that are specifically reactive to each [7]. While much research has focused on transmission, pathology, and diagnostics of URTD in *Gopherus* [5], understanding is still lacking as to the presence and importance of this disease across the geographic range of this genus, and the nature by which this disease may impact population viability. Given the intense research focus on this disease over multiple decades, it still remains an enigmatic and yet potentially devastating source of mortality in tortoises [8].

While *M*. *agassizii* and *M*. *testudineum* have been considered invasive pathogens, they are present across multiple sites within the range of Gopher Tortoises (*Gopherus polyphemus*). Diemer Berish et al.[9] found that evidence of URTD was present across many populations of Gopher Tortoises in central and northern Florida and [10] found that the spread of URTD is socially driven. McGuire et al. [11] sampled eleven populations of Gopher Tortoises in Georgia and found that evidence of URTD was present in seven of these sites. However, while [9] and [11] are the most extensive state-wide surveys of URTD in Gopher Tortoises, these two studies found relatively different epidemiological patterns of URTD as diagnosed by antibody titers to the pathogens. Specifically, [9] found that serologic tests of *Mycoplasma* were present at generally low levels within populations of Gopher Tortoises, while [11] found that populations typically had either very high or very low rates of seroprevalence, with very few sites intermediate in this test of disease prevalence. Additionally, [11] found that the rate of seroprevalence to *M*. *testudineum* was higher (73% of sites were seropositive) than previously reported rates of prevalence of this pathogen from sites in northeastern Florida (27%; [7]). As a result of the enigmatic nature of diagnostic tests, disease presence and mortality events, URTD in *Gopherus* has been described as context-dependent ([12]).

Gopher Tortoises have experienced extensive range-wide population declines ([13, 14]) and disease has exacerbated these declines [5]. Gopher Tortoises are currently listed as federally threatened in the western portion of the species’ range, west of the Mobile River in Alabama; moreover, the Gopher Tortoise is listed as a candidate species for federal protection throughout the remainder of its range ([15]). Populations in the eastern range of Gopher Tortoises, in Alabama and northwestern Florida, are considered peripheral, and therefore may be at an increased risk of population extinction ([16]). While intense conservation efforts are underway for Gopher Tortoises in Alabama, no systematic study has been conducted in this region of the species’ range to identify the nature of URTD in these important populations of tortoises.

Herein, our goals were to survey for the presence of URTD and its associated diagnostic tests in seven populations of Gopher Tortoises in Alabama. The seven populations include the largest populations of Gopher Tortoises on public lands in the state, and are thus important for long-term management and conservation efforts. Beyond simply assessing disease prevalence, we were also interested in testing the hypothesis that diagnostic tests of URTD are consistent with external disease symptoms. This goal provides a better understanding of the epidemiology of this disease, and provides a context for how to best monitor disease in free-ranging *Gopherus*.

## Materials and methods

### Study sites

**Fig 1:**
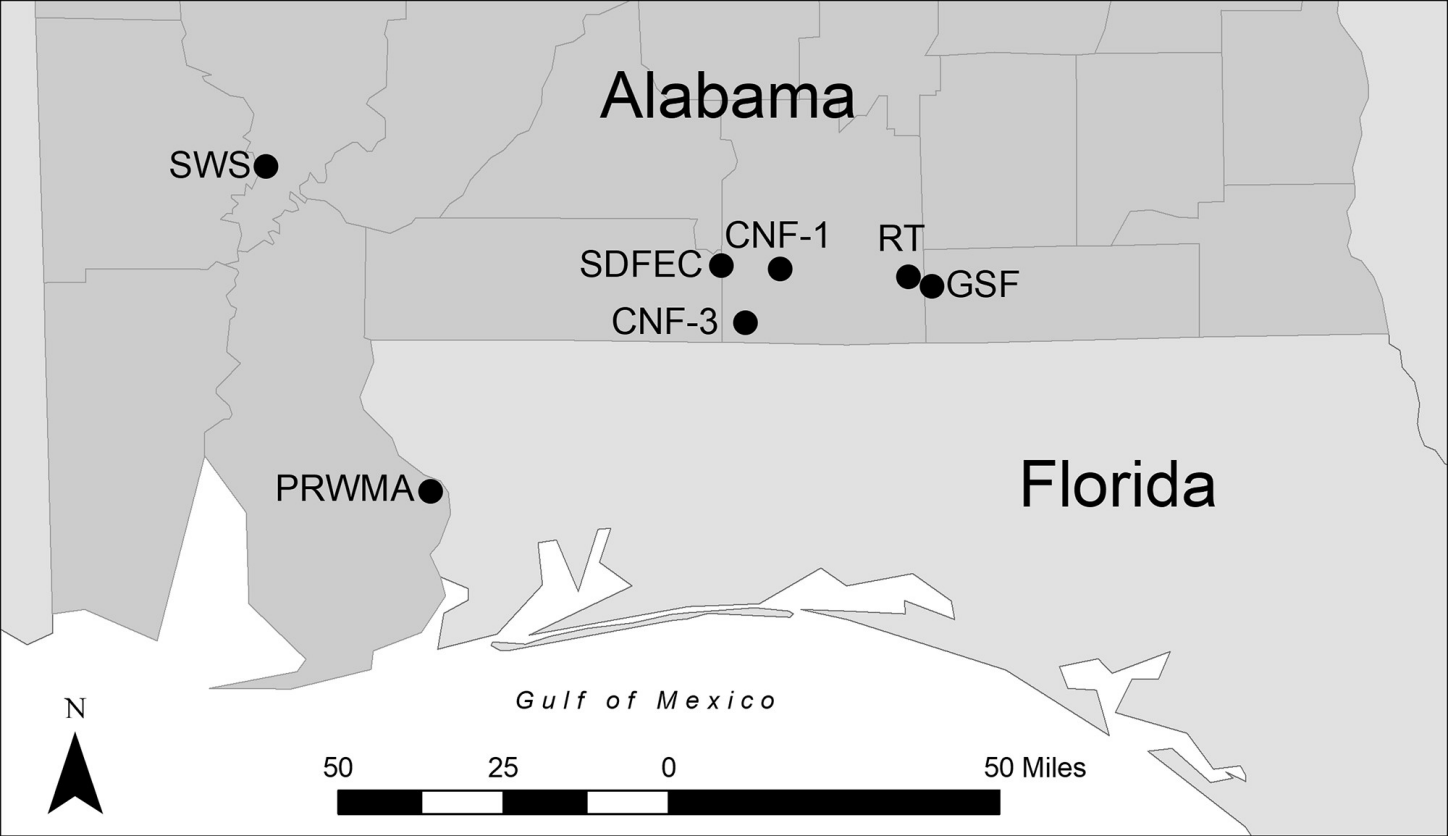
Study sites. Upper respiratory tract disease was sampled in populations of Gopher Tortoises from seven sites in Alabama. Counties in gray indicate Gopher Tortoise distribution in Alabama.

Tortoises were sampled at seven sites in Baldwin, Clarke, Covington, and Geneva counties of Alabama (Fig 1). Two of the sites are long-term Gopher Tortoise study sites and are located in Conecuh National Forest (Conecuh National Forest Site 1, CNF-1, and Conecuh National Forest Site 3, CNF-3). Three of the sites are located on state-owned lands that are maintained as wildlife management areas: Rayonier Tract, RT, and Geneva State Forest, GSF, which are both maintained under the Geneva Wildlife Management area, and Perdido River Wildlife Management Area, PWMA. One state-owned site, Stimpson Wildlife Sanctuary (SWS), is closed to the public and is maintained as a wildlife sanctuary. The seventh site, Solon Dixon Forestry Education Center (SDFEC), is privately owned by Auburn University School of Forestry and Wildlife Sciences. All seven sites are actively managed for multi-use, including timber production and wildlife management. Each site was sampled once in either 2013, 2014, or 2015, with the exception of SDFEC, which was sampled in all three years.

### Tortoise capture, sampling, and assays

Tortoises were trapped using custom-ordered Tomahawk live traps (Hazelhurst Wisconsin USA) placed at the entrance of each occupied burrow. Adult tortoises (> 18 cm straight mid-line carapace length) were targeted for this study. Traps were shaded using either a piece of burlap or vegetation from around the burrow, and traps were checked twice daily. In most cases, a blood sample (0.3–1.0 ml) was collected upon capture from the femoral vein of each tortoise using a 25 gauge needle affixed to a 1 ml syringe that had been previously heparinized with a sterile 0.5% sodium heparin solution diluted in ultrapure water (Sigma Aldrich, St. Louis Missouri USA). In the rare cases in which blood could not be collected from the femoral vein, whole blood was collected either from the brachial vein or subcarapacial venous sinus. A sample was not used in immune assays if it appeared to be contaminated with lymphatic fluid. Prior to venipuncture, the site was sanitized with an isopropyl alcohol wipe to prevent contamination or site infection. Whole blood samples were temporarily stored on ice, centrifuged and plasma separated and stored in liquid nitrogen within one hour of collection. Samples were thawed once prior to diagnostic submission to make serial aliquots of plasma samples for later assays. Immediately upon capture, tortoises were weighed and later measured for morphometric (e.g. relative condition) calculations.

Each tortoise was visually scored for external symptoms of URTD according to established guidelines [17]. Specifically, URTD symptoms were scored from 0 – 3 (none—severe) at five focal locations: nares, eyes, conjunctiva, eyelids, periocular region [17]. In addition to scoring active signs of disease (e.g. nasal drip, mucous build up or inflammation), the presence or absence of nasal scarring consistent with URTD was classified. This scarring included obvious nares asymmetries, erosion of the nares and/or erosion of the upper beak. The same person (JMG) scored all animals to reduce sampler bias in disease symptoms. Relative condition [18] was calculated by dividing each animal’s mass by its predicted mass. Predicted masses were calculated from the slope of an ordinary least squares regression between all ln-transformed lengths and ln-transformed masses.

Nasal lavage samples were collected following the guidelines outlined in [17] and per guidance from the staff at the University of Florida Mycoplasma Research Laboratory. Six milliliters of sterile saline were flushed through the paranasal sinuses using a sterile 22 gauge intravenous catheter, with the stylet removed, attached to a 10 ml sterile syringe. The lavage sample was collected as it dripped out of the nares into a sterile specimen collection cup. Immediately upon lavage collection, one milliliter of sterile SP4 *Mycoplasma* culture medium (University of Florida Mycoplasma Research Laboratory, Gainesville FL) was added to the sample. Lavage samples were immediately divided into aliquots in two milliliter cryogenic vials and were flash frozen in liquid nitrogen. Lavage samples were stored during each field season at -80 C and were never thawed; following each field season, all samples were sent as a batch to the University of Florida Mycoplasma Research Laboratory. Lavage samples were submitted for PCR/culture diagnostic assays of *M*. *agassizii* and *M*. *testudineum*. Plasma samples were also submitted to the University of Florida Mycoplasma Research Laboratory for diagnostic assays of antibodies to both *M*. *agassizii* and *M*. *testudineum*.

Following all data collection, tortoises were immediately released at the point of capture, thus tortoises were not held for a prolonged amount of time. Traps was decontaminated with a 10% bleach solution in between individuals and/or trap sites to minimize the risk of spreading disease among individuals. While the effects of trapping on acute stress responses are generally not well known for *G*. *polyphemus*, [19] found that trapping did not cause a glucocorticoid response in this species. Tortoises with external symptoms of disease were also immediately released at their point of capture, as we did not want to affect the demography of the tortoise populations in this study.

In addition to PCR/culture assays, the presence of *M*. *agassizii* and *M*. *testudineum* DNA was assessed using a quantitative PCR (qPCR) technique according to the protocol by [20]. This assay was run using *M*. *agassizii* and *M*. *testudineum* DNA as positive controls as well as one negative control (DNA free water) and validated to have similar specifications as published in [20,21]. Genomic DNA was extracted from 500 ul of the nasal lavage sample using a DNEasy blood and tissue extraction kit (Qiagen, Redwood City CA) and re-suspended in 100 ul of assay buffer. Quantitative PCR reaction conditions were according to [20]. It should be noted that samples were collected using a greater volume of saline and SP4 culture media (7 ml instead of 3 ml or 1 ml) than in [20] and [22]. Therefore, a sample was considered to be positive if any one (instead of two) of three triplicated samples had a Ct value of 40 or less. This choice may lead to an increased probability of false positives, but was considered appropriate in light of quantification of increased error and probability of false negatives in tortoise nasal lavage samples with low *M*. *agassizii* DNA concentrations (Ct values of 38–40) [21]. Repeated runs of the same sample on one plate (e.g. testing samples in triplicate) are primarily important in quantification of intra-assay variability [23]. Repeated testing (inter-assay variability) of Desert Tortoise and Gopher Tortoise samples with such low amplification rates at these high Ct values show repeatability of 1–2 amplifications per triplicate samples (unpublished data).

### Statistical analyses

Two different multiple logistic regressions were performed in R [24] to test if *M*. *agassizii* antibody titer, *M*. *testudineum* titer, or relative condition were predictive of either (1) active symptoms of URTD or (2) scarring consistent with past or chronic URTD. Thus, one regression was run with the above data as predictors of present disease symptoms, and a second regression was run with the above data as predictors of potentially past disease symptoms that caused scarring to the nares. Externally-visible URTD status or scarring was analyzed as a single response (either present or absent).

A G test was used to test the effects of year on antibody titer status to each pathogen at SDFEC, as well as to test the effects of sex on antibody titer. To test for a potentially subtler effect of disease status on relative condition, separate analyses were performed on tortoises that had at least one external symptom of URTD and used a Model II regression to compare relative condition to total disease score (by summing the totals of symptoms, i.e. 0–3, of all of the focal areas that were visually scored). Model II regression was used in this analysis because it does not assume zero variance in x variables (i.e., both variables in the regression analyses could be response variables to other fixed variables).

## Results

### Antibody presence across sites and disease markers

**Fig 2:**
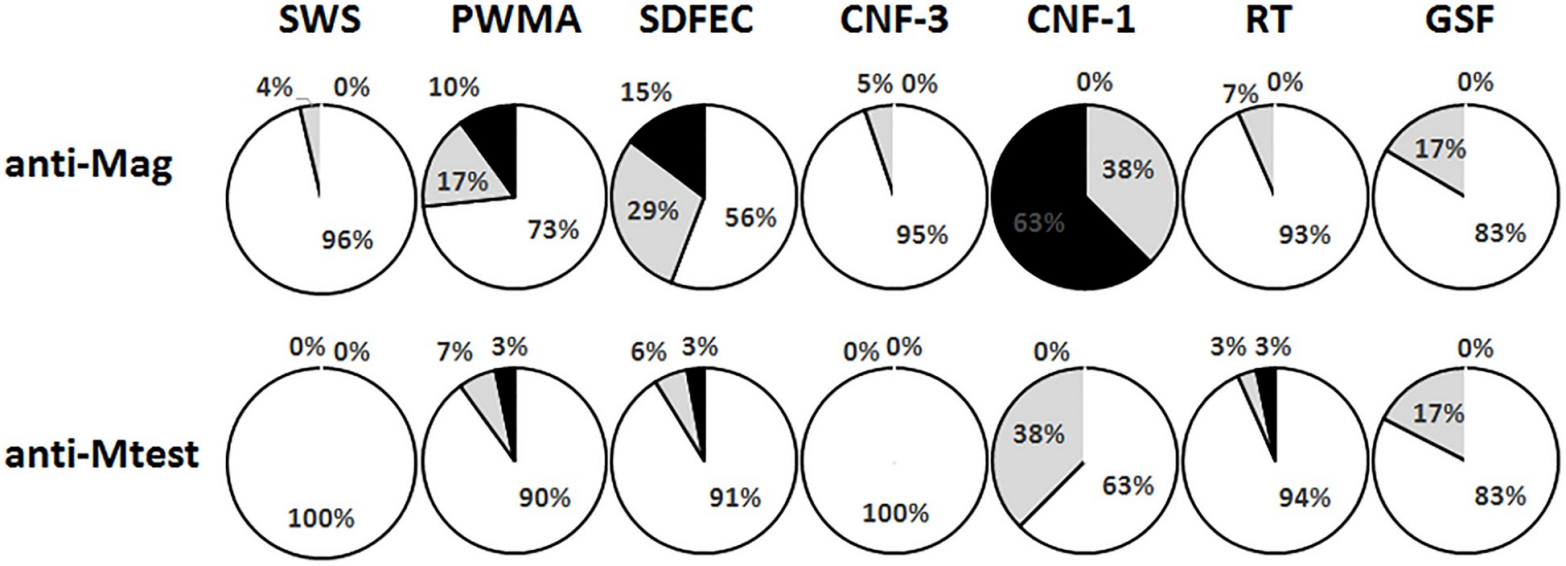
Results of *Mycoplasma* antibody titers in *Gopherus polyphemus* across seven sites in Alabama. Antibody titers to *Mycoplasma agassizii* (Mag) and *Mycoplasma testudineum* (Mtest) in *Gopherus polyphemus* varied across sites sampled in Alabama. Positive titers are indicated in black, suspect titers are indicated in gray and negative titers are indicated in white. Percentages were rounded to the integer, thus the total percentage at Site 1 exceeded 100% because 37.5% and 62.5% were both rounded up.

Every site had animals present that were at least suspect for exposure to *M*. *agassizii* (Table 1). The site with the highest frequency of animals with positive titers to *M*. *agassizii* was CNF-1, which was 63% positive and 37% suspect (Fig 2). Otherwise, prevalence of antibodies to *M*. *testudineum* was very low; in two sites, CNF-3 and SWS, 100% of the animals we sampled were negative for antibodies to this pathogen.

**Table 1:** URTD in Gopher Tortoise in Alabama separated by year, number of animals qPCR positive for *Mycoplasma agassizii*, number of animals with active symptoms of URTD, and number of animals with nasal scarring consistent with URTD.

Site	Year sampled	No. sampled	No. symptomatic (showing any external URTD symptoms)	No. with scarring	No. qPCR positive/total no. qPCR assayed for *M*. *agassizii*
**GSF**	2014	24	5	3	0/18
**RT**	2015	33	8	14	1/30
**CNF-1**	2013	24	2	1	0/12
**CNF-3**	2015	20	7	14	1/19
**SDFEC**	2013, 2014, 2015	39	13	16	2/33
**PWMA**	2015	29	7	17	0/26
**SWS**	2015	28	5	17	1/27

At SDFEC, which was sampled in three consecutive years, the prevalence of antibodies to *M*. *agassizii* varied significantly among sampling years (G-test: G^2^ = 36.88, d.f. = 2, P < 0.0001). Specifically, very high seroprevalence was found in 2013 (42% positive, 42% suspect, 16% negative), zero seroprevalence was found in 2014 (0% positive, 8% suspect, 92% negative) and low seroprevalence was found in 2015 (0% positive, 25% suspect, 75% negative).

Neither antibody titer nor relative condition significantly predicted the presence of disease symptoms consistent with URTD (Multiple logistic regression, n = 174: *M*. *agassizii* P = 0.365; *M*. *testudineum* P = 0.060; relative condition P = 0.176). However, a significant positive relationship was found between antibodies to *M*. *agassizii* and the presence of nasal scarring, but there was not a significant relationship between antibodies to *M*. *testudineum* and presence of nasal scarring (Multiple logistic regression, n = 174: *M*. *agassizii* P = 0.032; *M*. *testudineum* P = 0.391; relative condition P = 0.364).

No differences were found in antibody titers between males and females for either *M*. *agassizii* (G-test: G^2^ = 0.019, d.f. = 1, P = 0.89) or *M*. *testudineum* (G-test: G^2^ = 0.007, d.f. = 1, P = 0.93). Similarly, male and female *G*. *polyphemus* did not differ in the proportion of animals showing active disease symptoms (G^2^ = 0.0008, d.f. = 1, P = 0.97) or the presence of scarring consistent with chronic or past infection with URTD (G^2^ = 0.0007, d.f. = 1, P = 0.98).

**Fig 3:**
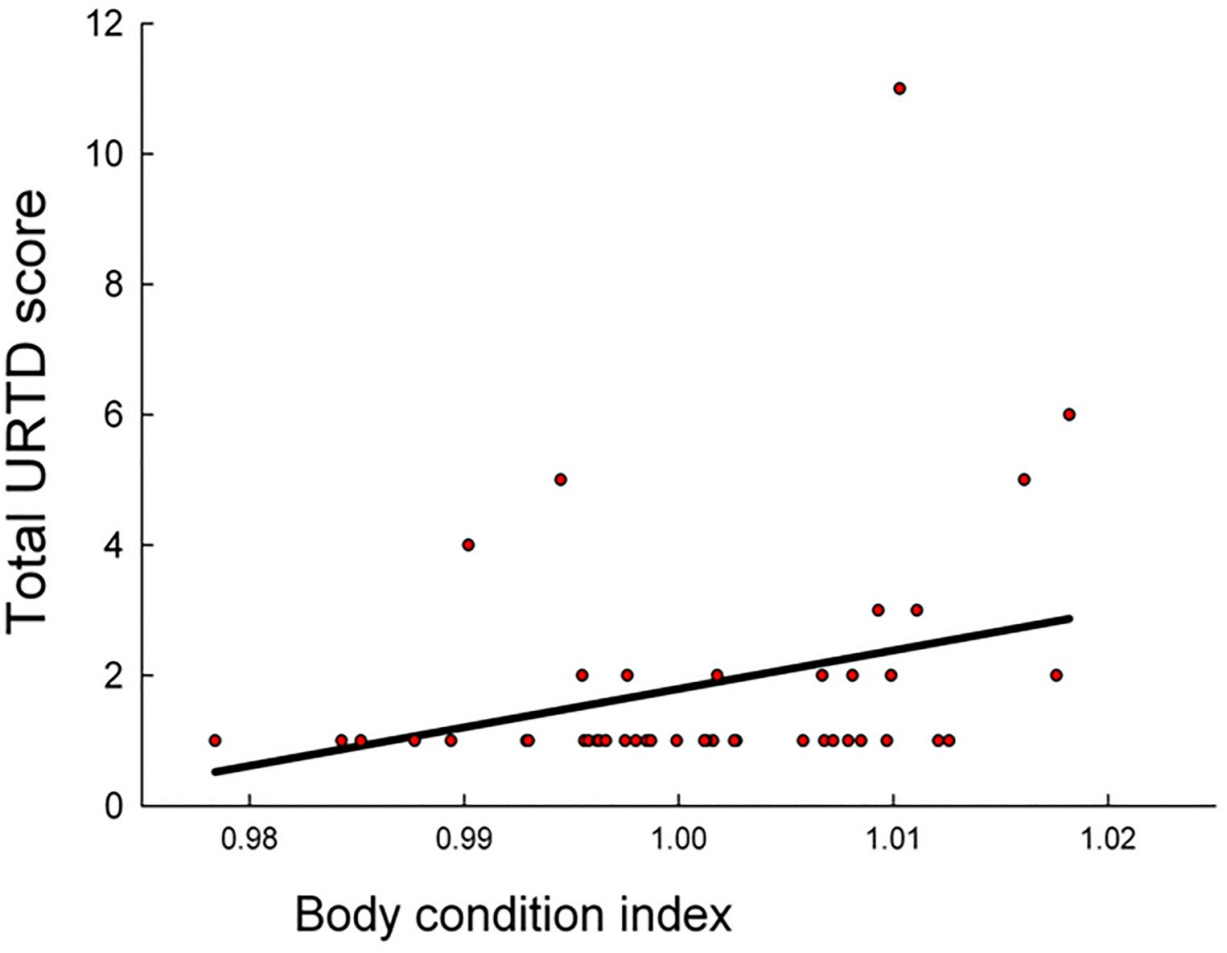
Relationship between relative condition and disease status in *Gopherus polyphemus*. A positive relationship was found between relative condition and total upper respiratory tract disease score in Gopher Tortoises from Alabama (Model 2 regression: P < 0.05, R^2^ = 0.084).

A positive relationship (Model 2 linear regression: P < 0.05, R^2^ = 0.084, Fig 3) was found between total disease score in animals with clinical signs of URTD and relative condition.

### Pathogen presence across sites

None of the lavage samples were culture positive for either *M*. *agassizii* or *M*. *testudineum*. However, the presence of *M*. *agassizii* DNA using the qPCR was identified at four of the sites (RT, CNF-3, SDFEC and SWS; Table 1 for pathogen survey results and sample sizes by site). For all positive samples, one out of three replicates amplified *M*. *agassizii* DNA, at a Ct of 38 or above, and therefore had low quantities of DNA in the collected samples of roughly 6 ml of saline/SP4 culture media. None of the animals sampled were qPCR positive for *M*. *testudineum*.

## Discussion

Evidence of URTD was present in all sampled Gopher Tortoise populations in Alabama further adding to the body of literature that indicates this disease geographically widespread, and that it may be of large-scale demographic importance to tortoise populations. However, these data found a weak link between the traditional diagnostic tests of *M*. *agassizii* and *M*. *testudineum* and external symptoms expressed by animals likely infected with URTD. In fact, no association between diagnostic tests for *M*. *testudineum* and active disease symptoms was found and, of these symptoms, only nasal scarring was significantly correlated with *M*. *agassizii* antibodies. We often found tortoises that possessed nasal scarring consistent with prior URTD (such as eroded or asymmetric nares), but did not possess external symptoms consistent with an active URTD (such as a nasal drip); because the *M*. *agassizii* antibody titers were predictive of scarring but not active URTD symptoms, it likely takes an extended amount of time for diseased tortoises to become scarred, mount an immune response (thus produce antibodies), and then potentially clear the infection, or at least reduce active signs of URTD (such as the nasal drip).

Significant variation was present in *M*. *agassizii* antibody titers among years at SDFEC. Previous research [25] had found a similar pattern in which seroprevalence to *M*. *agassizii* varies across years. An epidemiological model of URTD in tortoises has emerged that suggests entire populations are generally either positive or negative (e.g. [11]) for *Mycoplasma* antibodies. However, if that model was correct, the extreme inter-annual variation as we observed would not be predicted within diseased populations because the pathogens and associated antibodies are not likely cleared from all individuals over only a single year (e.g. [7]). Resampling the same individuals over time might help resolve this, although it is unlikely that the significant differences in antibody titers among the three years we sampled at SDFEC are due to random chance alone. These data demonstrate that caution should be applied when interpreting such specific diagnostic assays to infer populations’ disease status, as the antibody titers were widely variable among years within a population. This is especially true for populations that are only sampled within a single time point. Furthermore, as has been previously suggested [26], because of the diversity of potential pathogens of URTD, populations should not be considered disease “positive” based solely on the presence of *Mycoplasma* antibodies.

*Mycoplasma agassizii* DNA was isolated from four of the seven sites using a qPCR technique. Interestingly, the site with the highest antibody prevalence to *M*. *agassizii* was among the sites from which this pathogen’s DNA was not found. Furthermore, *Mycoplasma* DNA was isolated from the three sites with the lowest seroprevalence to *M*. *agassizii* (SWS, CNF-3 and RT). Many of the extant tortoise populations in Alabama are relatively small (15–30 individuals), thus while larger sample sizes may clarify patterns between pathogen presence and seroprevalence, many sites simply lack adequate numbers of tortoises to further resolve this discrepancy.

*Mycoplasma testudineum* has been considered the less pathogenic of two known URTD pathogens in *Gopherus* [27]. Data in this study suggest that this pathogen is seropresent in at least three (e.g. PWMA, SDFEC and RT) and maybe five (including two suspect sites: GSF and CNF-1) sites in Alabama. Data from [11] suggested that co-occurrence of both *M*. *agassizii* and *M*. *testudineum* may increase the risk for tortoises to develop URTD; however, [17] found that there was no relationship between co infection of both pathogens and URTD in several *Gopherus* species, including *G*. *polyphemus*. We commonly observed animals with symptoms of URTD, yet both pathogens were detected very infrequently using the traditionally used diagnostic tests of URTD. This may further indicate that too much focus has been placed on only a few potential pathogens of URTD, and that other pathogens may likely be more important agents of morbidity and mortality in Gopher Tortoises.

In Georgia, [11] found that almost half of the sampled Gopher Tortoise populations had individuals that we seropositive for *M*. *agassizii*, a pattern which was very similar to that which we found in Alabama, in which three of seven sites had tortoises that were seropositive to *M*. *agassizii*. However, [11] found that 73% of sampled populations had tortoises that were seropositive to *M*. *testudineum*, but we found that only three out of seven sites were antibody positive to this pathogen. We found that two out of seven sites were antibody positive for both *Mycoplasma* species, while [11] found that 45% of sampled sites were seropositive to both. Given that the sampling methodology for antibody testing was the same between McGuire et al.’s [11] study and this study, the combined data may indicate a regional pattern by which *Mycoplasma* may be either more prevalent and/or of greater importance to eastern populations of Gopher Tortoises. Additionally, our furthest west population, SWS, had the lowest seroprevalence to *M*. *agassizii* (e.g. 4% suspect, 0% positive) and had no seroprevalence to *M*. *testudineum*. This population is very near the federally threatened and genetically distinct western population of Gopher Tortoises [28]. As Gopher Tortoise habitat gets progressively less suitable near the species’ northwestern range limit, populations get more socially isolated, and experience greater demographic constraints [16]; thus, socially-mediated pathogens (such as *Mycoplasma*, [10]) may also become less abundant. As described, this pattern applies only to *Mycoplasma* antibody prevalence, because we detected one individual that was qPCR positive to *M*. *agassizii*, five out of 28 individuals that had external symptoms of URTD, and 17 out of 28 individuals that had scarring consistent with URTD. In a recent URTD-associated Gopher Tortoise die off in central Florida, [8] found that while seropositive animals were more likely to express symptoms of URTD, 32% of sampled animals were seronegative, but still had visible symptoms of URTD. This lack of clarity between URTD symptoms, antibody prevalence, and pathogen DNA prevalence further highlights the complex nature of surveillance for this disease. One possible explanation for such variability between disease symptoms and *Mycoplasma* antibodies may be that another causative pathogen of URTD renders the upper respiratory tract more susceptible to a secondary *Mycoplasma* infection. Alternatively, in closely-related desert tortoises (*G*. *agassizii*), antibody responses to a protein (chicken ovalbumin) which mimics the immune response to bacteria have been shown to occur over months and persist for more than a year [29]. Both these long time frames to peak antibody responses and unexplained persistence of antibodies post-immune challenge are expected to create some disconnect between immune responses and pathogen presence and/or disease status.

It has been suggested that other pathogens (such as pathogenic viruses, [5]) may be important sources of disease in North American tortoises. Given that we found the disease diagnostic assays for *M*. *agassizii* and *M*. *testudineum* were not good indicators of current disease state, our data suggest that other undetected pathogens may be important agents of URTD in Gopher Tortoises. Further research should focus on identifying additional pathogens of URTD, as well as focus on pathogens that may be more important in driving rapid population die offs observed elsewhere in the range of Gopher Tortoises (e.g., [8,30]).

A negative relationship was expected between relative condition and URTD severity. However, a significant positive relationship was observed between these two parameters. We had predicted a negative relationship between relative condition and disease because we expected that either poor-condition animals become susceptible to disease, or that diseased animals are sickened to poorer condition. One potential interpretation of this positive relationship is related to either increased food or water retention in diseased animals. Tortoises are hindgut-fermenting herbivores, thus processes that reduce digestive passage rates (causing potentially increasing digesta retention) might increase parameters of condition (such as relative mass), even if an animal is actually in a poorer physiological state. Hind-gut fermenting mammals have been shown to reduce digestive efficiency in response to heat stress [31], although, to our knowledge, no studies have specifically addressed mass gain in diseased hind-gut fermenting herbivores. It is possible that in response to the physiological stress of disease, digestive passage rates are reduced thereby increasing condition parameters. It is also possible that true relative condition is increased in response to disease, as a compensatory mechanism in animals fighting infection. We do not have the ability to fully address this problem with our data, but future studies should investigate the response of condition to infection in this taxon.

Sandmeier et al. [12] suggested that URTD in *Gopherus* is context dependent and may be exacerbated by environmental factors that render populations more susceptible to disease. Furthermore, [32] demonstrated that winter reduces constitutive humoral immunity in Gopher Tortoises, [33] demonstrated that thermal instability, regardless of season, negatively affects immunity in Gopher Tortoises. Experimental studies such as these can specifically address how global change may act in negative synergy with pathogens to threaten species of conservation concern [1]. As experimental ecoimmunology continues to emerge as a field, we may further identify causative mechanisms by which disease prevalence is altered in wild species, beyond simply assessing presence of potential pathogens within populations. This more nuanced approach, by which the immune system is examined as an integrated response of both pathogen and the environment, may better inform currently unclear patterns between pathogen presence and disease severity in wild populations.

## Supporting information

S1 File Data used in analyses for this study. (XLSX)
